# Tanshinone IIA Protects against Dextran Sulfate Sodium- (DSS-) Induced Colitis in Mice by Modulation of Neutrophil Infiltration and Activation

**DOI:** 10.1155/2016/7916763

**Published:** 2016-01-03

**Authors:** Xiaowei Liu, Haiyue He, Tingting Huang, Zhen Lei, Fuquan Liu, Guangyu An, Tao Wen

**Affiliations:** ^1^Department of Digestive Diseases, Second Xiangya Hospital, Central South University, Changsha, Hunan 410013, China; ^2^Medical Research Center, Beijing Chao-Yang Hospital, Capital Medical University, Beijing 100020, China; ^3^Department of Intervention Therapy, Beijing Shijitan Hospital, Capital Medical University, Beijing 100038, China; ^4^Department of Oncology, Beijing Chao-Yang Hospital, Capital Medical University, Beijing 100020, China

## Abstract

Neutrophils play a critical role in the initiation and maintenance of intestinal inflammation. However, conventional neutrophil-targeted therapies can impair normal host defense. Tanshinone IIA has been recently revealed to act directly on neutrophils. Hence, we aimed at investigating whether Tanshinone IIA can protect against experimental colitis through modulation of neutrophils. We induced colitis in C57BL/6 mice by giving 3% dextran sulfate sodium (DSS) orally, and meanwhile, we treated mice daily with Tanshinone IIA intraperitoneally. The severity of colitis was evaluated by calculating disease activity index (DAI) and histological parameters. Neutrophil infiltration and activation in the colons of mice were measured. Moreover, whether Tanshinone IIA has direct effects on neutrophil migration and activation was determined in vitro. Our data showed that Tanshinone IIA significantly ameliorated the severity of DSS-induced colitis in mice, evidenced by the reduced DAI and improved colonic inflammation. In addition, Tanshinone IIA decreased neutrophil infiltration of intestinal mucosa and activation and reduced colonic inflammatory cytokines in DSS-treated mice. Furthermore, Tanshinone IIA was demonstrated to significantly suppress neutrophil migration and activation. These results provide compelling evidence that Tanshinone IIA has a therapeutic potential for alleviating inflammatory colitis in mice, which is possibly mediated by the immunomodulation of neutrophils.

## 1. Introduction

Ulcerative colitis (UC), a major form of inflammatory bowel diseases (IBD), is a chronic and relapsing inflammatory disorder of the colorectum that results from an abnormal interaction between colonic microflora and mucosal immune cells in a genetically susceptible host [1–3]. The etiology and pathogenesis of UC have not been fully elucidated. Recent studies suggest that transmural infiltration of leukocytes contributes mostly to the initiation and maintenance of intestinal inflammation and subsequent mucosal disruption and ulceration [4, 5]. Neutrophils, the major infiltrating inflammatory cells, serve as a first-line defense against invading microorganisms or tissue injury, leading to protection of the human body against these insults [6, 7]. Significant neutrophil infiltration of the intestinal mucosa has been always observed in both human and murine colitis, including neutrophil migration across intestinal epithelia, neutrophil accumulation in the inflamed intestine, release of massive amounts of reactive oxygen species (ROS), and overproduction of inflammatory cytokines [8]. However, excessive or persistent neutrophil infiltration is disadvantageous and participates in the pathogenesis of various inflammatory diseases including UC. Clearance of tissue neutrophils is essential for resolution of inflammation and for the maintenance of tissue homeostasis [9]. It was reported that neutrophil depletion reduced disease severity in experimental models of colitis, which supports a key pathogenic role for neutrophils [10, 11]. However, this approach remains partially successful and may have side effects such as impairing host defense against infection. Thus, exploring new strategies to modulate neutrophil infiltration or activation without altering their normal host-protective functions may provide attractive therapies to UC.

**Figure 1:**
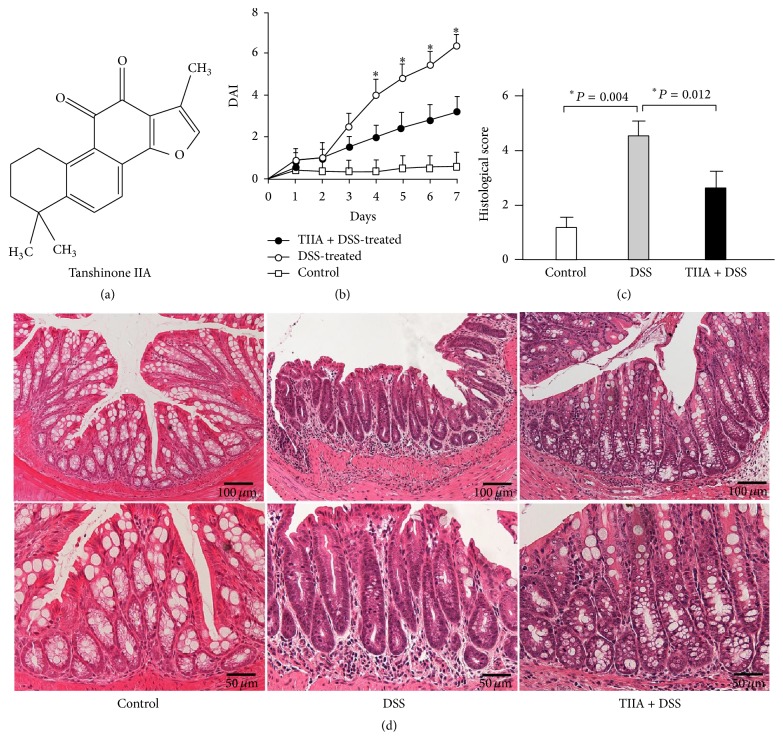
Protective effects of Tanshinone IIA against DSS-induced colitis in mice. (a) Chemical structure of Tanshinone IIA. (b) Clinical DAI of DSS-treated mice based on diarrhea, fecal occult blood, and rectal prolapse (mean ± SD, *n* = 5 mice/group). ^*∗*^ *P* < 0.05 indicates a significant difference between DSS-treated and Tanshinone IIA + DSS-treated mice. (c) Histological scores of colon inflammation in DSS-treated mice or Tanshinone IIA + DSS-treated mice (mean ± SD, *n* = 6 mice/group). (d) Representative images of HE-stained colonic tissues from colitis mice treated with DSS or Tanshinone IIA + DSS. Data are representative of at least 3 experiments.

Tanshinone IIA (Figure 1(a)) is a natural extract isolated from* Salviae miltiorrhizae*, a Chinese medicinal herb traditionally used to treat cardiovascular disorders, Alzheimer's disease, and liver fibrosis [12, 13]. Tanshinone IIA is known to possess anti-inflammatory and antioxidative activities and has exhibited protective effects in various inflammatory conditions [13, 14]. Of particular interest, a recent study by Robertson et al. reported that Tanshinone IIA has pronounced effects in inducing neutrophil apoptosis and promoting neutrophil reverse migration [12]. Given the etiological role of neutrophils in the intestinal inflammation, we intended to investigate whether Tanshinone IIA has protective roles against experimental colitis via modulating neutrophil functions.

In this study, we demonstrate that Tanshinone IIA can ameliorate dextran sulfate sodium- (DSS-) induced colitis in mice. The beneficial effects of Tanshinone IIA are achieved by suppressing migration and activation of neutrophils in inflamed tissues and downregulating the production of proinflammatory cytokines. Our studies suggest that Tanshinone IIA may be a new therapeutic agent for colitis.

## 2. Material and Methods

### 2.1. Animals

Male C57BL/6 mice, aged 8–10 weeks, were obtained from the Laboratory Animal Center of Capital Medical University (Beijing, China). The mice were maintained in standard housing cages under specific pathogen free conditions. All experimental procedures were reviewed and approved by the Capital Medical University Animal Care and Use Committee and were in accordance with the institutional guidelines for the Care and Use of Laboratory Animals.

### 2.2. Experimental Design for Induction of Colitis and Drug Intervention

To induce colitis, DSS (40 kDa, Sigma Aldrich, USA) was dissolved in sterile water at a final concentration of 3% and presented to mice as drinking water for 7 consecutive days [15]. Negative control animals received water only. In another set of experiments, Tanshinone IIA (Sigma Aldrich, USA) was dissolved in DMSO and given to mice intraperitoneally daily at a dose of 200 mg/kg for 7 days during the colitis induction. Control mice received the same dose of vehicle only. Doses of Tanshinone IIA were selected based on our preliminary experiments. The disease activity index (DAI) was assessed daily during the course of treatments, which was calculated by scoring changes in animal weight, the presence of fecal blood/rectal bleeding, diarrhea, and mortality [16]. Mice were sacrificed on the 8th day and colons were removed for further analysis.

### 2.3. Histological Analysis

For histological analysis, the colons from mice were fixed in 10% neutral buffered formalin, processed, and embedded in paraffin. 5 *μ*m thick tissue sections were stained with hematoxylin and eosin (HE) using standard techniques. Histologic severity of colitis was assessed in a blinded fashion using an established histologic scoring system: (a) epithelial damage (0 points = none, 1 point = minimal loss of goblet cells, 2 points = extensive loss of goblet cells, 3 points = minimal loss of crypts and extensive loss of goblet cells, and 4 points = extensive loss of crypts) and (b) inflammatory cell infiltration (0 points = none, 1 point = infiltration around crypt bases, 2 points = infiltration in the mucosa, 3 points = extensive infiltration in the mucosa with edema, and 4 points = the submucosa infiltration) [17]. The total score ranged from a minimum of 0 to a maximum of 8. Scoring was done by two investigators who were blind to the treatment groups.

### 2.4. Assay for Intestinal Barrier Integrity

Intestinal barrier integrity was measured as described previously [16, 17]. In brief, mice were administered 200 *μ*L of FITC-dextran (molecular weight, 4 kDa; Sigma-Aldrich) at 600 mg/kg body weight by gavage. Blood was collected 4 h later by retroorbital bleeding. The serum concentration of the FITC-dextran was determined using a fluorimeter (excitation, 490 nm; emission, 530 nm). Serially diluted FITC-dextran was used to generate a standard curve. Animals were sacrificed immediately after bleeding, and colonic cryosections were prepared for fluorescence microscopy examination.

### 2.5. Determination of Colonic Infiltrating Neutrophils

For the detection of infiltrated neutrophils, colons were dissected from mice and fixed in 4% paraformaldehyde at 4°C overnight, followed by cryoprotection in 20% sucrose, and then embedded in a mixture of OCT compound and tissue freezing medium. 8 *μ*m thick cryosections were stained with rat anti-mouse Ly6G (2 *μ*g/mL, BD Biosciences) overnight at 4°C and were then incubated with FITC-conjugated donkey anti-rat IgG (1 : 200, Invitrogen) for 1 h at room temperature. These sections were mounted with Vectashield mounting medium (Vector Laboratories) and analyzed by a fluorescent microscope.

MMP-8 (matrix metalloproteinase-8) is a neutrophil-specific collagenase that aids in neutrophil tissue penetration and recruitment [11, 18]. So we performed additional measurement of neutrophil infiltration by employing western blot analysis of MMP-8 expression in the colon of mice. Briefly, frozen colonic tissues were weighed and homogenized in lysis buffer containing 50 mM Tris-HCl, 150 mM NaCl, 0.1% sodium dodecyl sulfate (SDS), 0.5% sodium deoxycholate, and cocktail protease inhibitors. Homogenates were centrifuged at 12,000 ×g at 4°C for 20 min and the supernatants were collected and stored at 80°C. The protein concentrations of the supernatants were determined using the Bradford method. Aliquots of 20 mg proteins were fractionated on 12% polyacrylamide-SDS gel. After electrophoretic separation, proteins were transferred onto PVDF membrane. The membrane was blocked with Tris-HCl Buffered Saline (TBS) containing 5% nonfat milk, followed by incubation with primary antibodies of MMP-8 (1 : 1000, abcam) or *β*-actin (1 : 1000, abcam). Then the membrane was treated with horseradish peroxidase-conjugated secondary antibody (1 : 2000, abcam). Antibody binding was visualized with an ECL chemiluminescence system (Pierce Biotech Inc., IL, USA) and short exposure of the membrane to X-ray films. The obtained signals were quantified using an image scanner (Bio-Rad Biotech, CA, USA) and an image analysis software (Bio-Rad Biotech, CA, USA).

### 2.6. Determination of Neutrophil Activation

We quantified the extent of neutrophil activation by measuring MPO activity and ROS production in the colon of mice, respectively. Briefly, colons were dissected, rinsed with cold saline, and cut into small pieces. Samples were homogenized in 50 mM phosphate buffer and centrifuged at 12,000 ×g at 4°C for 20 min. We assayed MPO activity, an enzyme occurring nearly exclusively in neutrophils, using a commercial kit (BioVision, CA, USA), according to the manufacturer's recommended protocol. Additionally, we determined the levels of ROS in the colonic homogenates by using the Intracellular ROS assay kit (Cell Biolabs, San Diego, CA, USA), according to the manufacturer's instructions. Briefly, the lysates were incubated with 50 *μ*M 2′,7′-dichlorodihydrofluoresceindiacetate (DCFH-DA) at 37°C for 1 h. Fluorescence intensities were measured by a fluorescence plate reader at 480 nm excitation/530 nm emission.

### 2.7. Measurement of Colonic Cytokines

We quantified IL-1*β*, IL-6, IL-10, and TNF-*α* levels in colonic homogenates of mice using the ProcartaPlex Multiplex Immunoassay (Luminex) on a Bio-Plex 200 system with the Bio-Plex Manger 5.0 software, according to the manufacturer's protocol.

### 2.8. Neutrophil Isolation and In Vitro Migration Analysis

The peripheral blood from mice was collected in 5 mM EDTA-coated tubes by cardiac puncture. After lysis of red blood cells, neutrophils were isolated by the Ficoll gradient centrifugation method [19]. Neutrophil purity was assessed using Wright-Giemsa staining and was found to be more than 95%.

Purified neutrophils were applied in migration assay as described previously [19]. Briefly, a total of 5 × 10^5^ neutrophils were placed in the top chamber of transwells (5 *μ*m pore size filter; Corning Inc., Corning, NY, USA) and CXCL1 (250 ng/mL; R&D Systems) was added to the bottom wells. RMPI 1640 medium without CXCL1 was used as a negative control. Tanshinone IIA was dissolved in DMSO and added to neutrophils with a final concentration of 10 mg/mL. Transwells were then incubated for 2 h at 37°C with 5% CO_2_. Neutrophils migrated into the bottom wells were imaged under a microscope and enumerated using a hemocytometer.

### 2.9. In Vitro Neutrophil Activation Assay

Neutrophils were pretreated with or without LPS (1 *μ*g/mL) in DMEM with 10% FBS for 1 h at 37°C with 5% CO_2_. Meanwhile, Tanshinone IIA was dissolved in DMSO and added to neutrophils with a final concentration of 10 mg/mL. The cells after treatment were harvested and homogenized in a lysis buffer. Then the homogenates were centrifuged at 10,000 ×g at 4°C for 20 min, and the supernatants were analyzed for MPO activity and multiple cytokines (IL-1*β*, IL-6, IL-10, and TNF-*α*) as described above.

In addition, ROS production in neutrophils was measured using DCFH-DA (Cell Biolabs, San Diego, CA, USA) as a fluorescence probe by flow cytometry, following the manufacturer's instructions. Briefly, the treated neutrophils were harvested and incubated with 10 *μ*M DCFH-DA for 30 min in the dark at 37°C. After washing with PBS, the cells were analyzed by flow cytometry (BD, FACS Calibur, San Jose, CA, USA) for ROS production.

### 2.10. Statistical Analysis

All values were expressed as mean ± standard deviation (SD). One-way ANOVA was used to assess differences between groups. Results were considered statistically significant when *P* < 0.05. All statistical analyses were calculated using Graph Pad Prism.

## 3. Results

### 3.1. Tanshinone IIA Alleviates the Severity of DSS-Induced Colitis in Mice

In this study, we used a murine model challenged with DSS to examine whether Tanshinone IIA could prevent intestinal inflammation. After 7-day oral administration of DSS, the mice developed significant intestinal inflammation, manifested by drastic weight loss, bloody diarrhea, and shortening of the colon, as compared to the control mice receiving water alone, which did not show any signs of inflammation. Notably, the intraperitoneal treatment with Tanshinone IIA markedly reduced the clinical scores of the DSS-induced colitis in mice (DSS + Tanshinone IIA) compared to the vehicle control (DSS + vehicle), which included less body weight loss, less diarrhea, or less fecal bleeding (Figure 1(b)). Histological analysis of colon showed that DSS induction resulted in typical inflamed signs, characterized by significant epithelial destruction, mucosal ulcerations, goblet cell loss, and infiltration of massive inflammatory cells, as compared to the non-DSS-treated mice. In contrast, treatment with Tanshinone IIA caused a remarkable improvement in histological scores, evidenced by the moderate inflammatory infiltrates with only focal mucosal erosion in mice (Figures 1(c) and 1(d)).

### 3.2. Tanshinone IIA Improves Intestinal Permeability in DSS-Treated Mice

**Figure 2:**
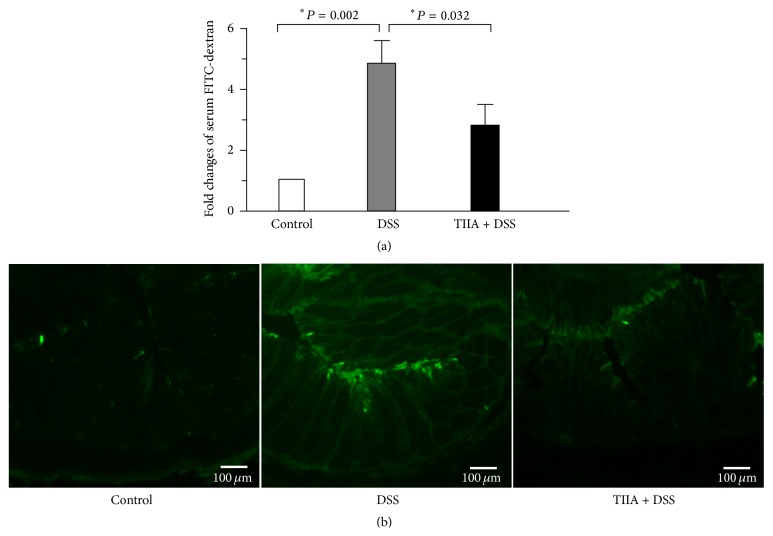
Protective effects of Tanshinone IIA on intestinal permeability in DSS-treated mice. (a) Serum concentrations of the FITC-dextran of DSS-treated mice were measured 4 h after oral administration of FITC-dextran (mean ± SD, *n* = 6 mice/group). (b) Representative images of fluorescent microscopic analysis of colonic cryosections in DSS-treated or Tanshinone IIA + DSS-treated mice 4 h after oral administration of FITC-dextran. Scale bars: 100 *μ*m. Data are representative of at least 3 experiments.

Since impaired intestinal permeability is an important feature during the course of DSS-induced colitis, we tested whether Tanshinone IIA affected intestinal permeability in mice. We fed the mice with FITC-dextran by gavage, and 4 h later we measured their serum levels of FITC-dextran to evaluate intestinal permeability. It showed that serum levels of FITC-dextran in DSS-treated mice were significantly higher than in the control mice, whereas cotreatment with Tanshinone IIA caused a remarkable decrease in serum levels of FITC-dextran (Figure 2(a)). Fluorescent microscopic analysis of intestinal cryosections in mice revealed no fluorescent infiltration of FITC-dextran in the control mice, whereas a higher level of fluorescent intensity was observed in the colonic tissues of DSS-treated mice. In contrast, the colonic tissues of Tanshinone IIA + DSS-treated mice showed a markedly decreased level of fluorescent intensity (Figure 2(b)). Collectively, these data indicated that DSS treatment caused the breached mucus integrity in mice, leading to increased intestinal barrier permeability, whereas Tanshinone IIA improved intestinal permeability in DSS-treated mice, thus providing protection against inflammation.

### 3.3. Tanshinone IIA Reduces Intestinal Neutrophil Infiltration

**Figure 3:**
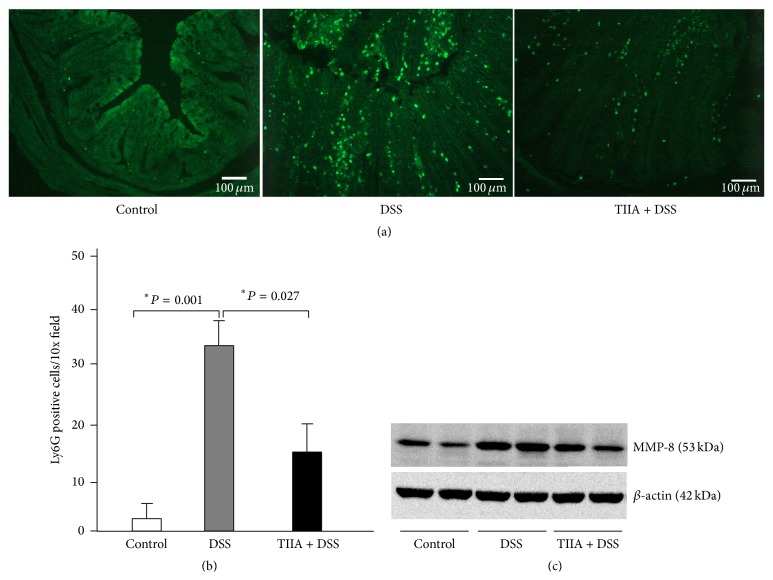
Tanshinone IIA reduces intestinal neutrophil infiltration. (a) Representative images of Ly6G positive lymphocyte infiltrates in the colonic sections of DSS-treated or Tanshinone IIA + DSS-treated mice. Scale bars: 100 *μ*m. (b) Positive staining areas were quantified per high-powered microscopic field (10x) based on six sections from three independent mice from each group. Error bars indicate mean ± the SD. (c) Expression of MMP-8 in colonic tissues of DSS-treated mice measured by western blots. *β*-actin was used as a loading control. The data represent at least three experiments.

To characterize whether neutrophil is closely involved in mediating DSS-induced colitis, we analyzed neutrophil infiltration of the mucosa. To this end, we first performed immunofluorescence staining of Ly6G, a specific marker for neutrophils, in colon cryosections of mice. Compared to the control mice that received water alone, there was a drastic increase of Ly6G positive cells around the mucosal area of DSS-treated mice, indicative of prominent neutrophil infiltration. In contrast, Tanshinone IIA + DSS-treated mice had less Ly6G^+^ cells in the mucosal tissues (Figures 3(a) and 3(b)). Additionally, we analyzed the expression of MMP-8 by western blotting to further assess neutrophil infiltration in the colonic tissues of mice. It showed that DSS induction caused significant expression of colonic MMP-8 in mice as compared to the controls, whereas MMP-8 expression was markedly lower in colonic tissues of Tanshinone IIA + DSS-treated mice (Figure 3(c)). Taken together, it suggested that neutrophil infiltration occurred during the course of DSS-induced colitis, while Tanshinone IIA significantly reduced neutrophil infiltration as evidenced by decreased expression of Ly6G and MMP-8, respectively.

### 3.4. Tanshinone IIA Suppresses Intestinal Neutrophil Activation

**Figure 4:**
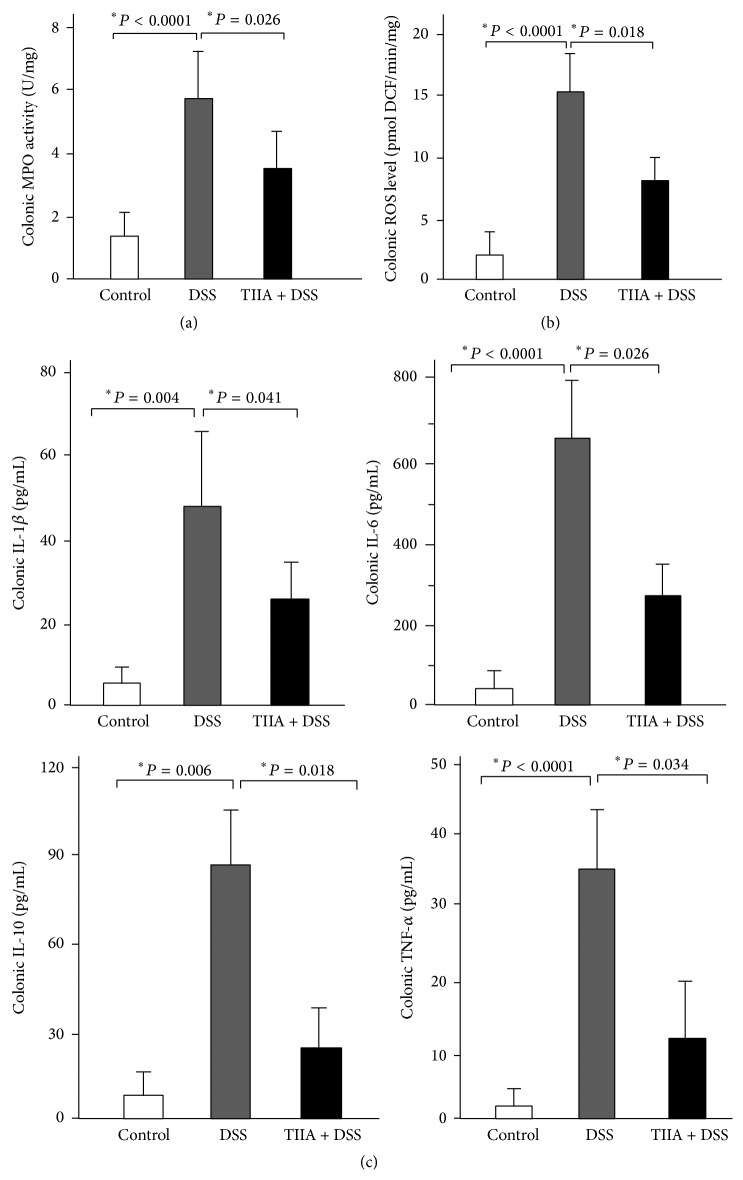
Inhibitory effects of Tanshinone IIA on intestinal neutrophil activation. (a) Colonic MPO activity in DSS-treated mice. (b) ROS levels in colonic tissues of DSS-treated mice. (c) The levels of inflammatory cytokines in colonic tissues. DSS remarkably increased MPO, ROS, and the four tested inflammatory cytokine levels in the colonic tissue of mice, whereas Tanshinone IIA significantly reduced their levels in mice (mean ± SD, *n* = 5-6 mice/group).

We also analyzed colonic MPO activity, a specific marker for neutrophil activation, in DSS-treated mice. It showed that MPO activity was remarkably increased after administration of DSS to mice, indicative of a significant activation of tissue neutrophils, whereas MPO activity was much lower in Tanshinone IIA + DSS-treated mice (Figure 4(a)), suggesting that Tanshinone IIA inhibited neutrophil activation. It is accepted that neutrophil activation leads to the release of ROS and the production of inflammatory cytokines, both of which are implicated in intestinal inflammation. Hence, we measured the levels of ROS and the inflammatory cytokines (IL-1*β*, IL-6, IL-10, and TNF-*α*) in the colonic tissues of mice. It showed that the levels of ROS and these cytokines were all increased significantly in DSS-treated mice as compared to the controls, whereas Tanshinone IIA suppressed the levels of ROS (Figure 4(b)) and the cytokines (Figure 4(c)) in colonic tissues of DSS-treated mice, thus presenting more evidence that Tanshinone IIA can inhibit neutrophil activation.

### 3.5. Tanshinone IIA Blocks Neutrophil Migration and Inhibits Neutrophil Activation In Vitro

**Figure 5:**
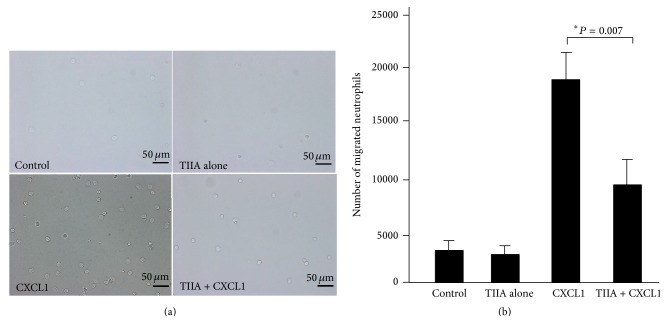
Effect of Tanshinone IIA on neutrophil migration in vitro. (a) Peripheral blood neutrophils were isolated and plated in a transwell system and cells migrated in response to CXCL1 at the bottom well were enumerated. Representative light images of neutrophils migrated into bottom wells. Scale bars: 50 *μ*m. (b) Quantitative measurements of migrated neutrophils. Results were representative of 3 independent experiments.

**Figure 6:**
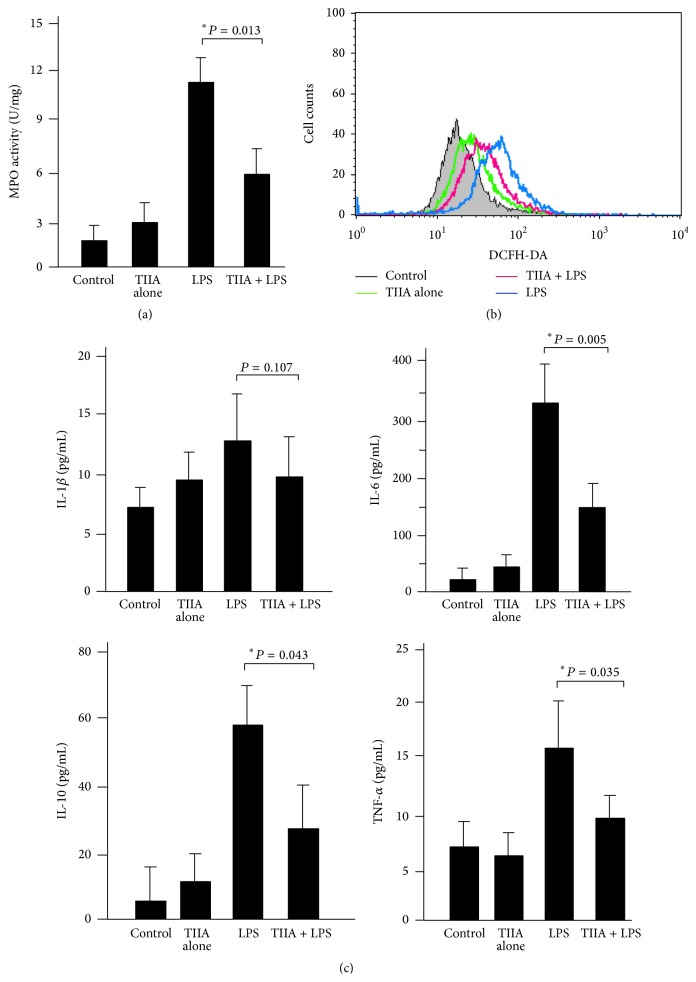
Effect of Tanshinone IIA on neutrophil activation in vitro. (a) The LPS-stimulated neutrophils had a significant increase in MPO activities, whereas coincubation with Tanshinone IIA markedly inhibited MPO activities. (b) The LPS-stimulated neutrophils produced high levels of ROS, which was determined by flow cytometry, whereas coincubation with Tanshinone IIA markedly inhibited ROS levels. (c) The LPS-stimulated neutrophils produced higher levels of cytokines (IL-6, IL-10, and TNF-*α*), whereas coincubation with Tanshinone IIA markedly inhibited the production of these cytokines. Results were representative of 3 independent experiments.

We next studied whether Tanshinone IIA-mediated protection was mainly dependent on its inhibitory action on neutrophils. We isolated mouse neutrophils and incubated cells in the presence or absence of Tanshinone IIA. First, the neutrophils were stimulated with CXCL1 to measure cell migration, and as expected the results showed that Tanshinone IIA had a significantly inhibitory effect on CXCL1-induced neutrophil migration (Figure 5). Second, the neutrophils were treated with LPS for 60 min to induce their activation, as assessed by determination of MPO activity and ROS release, as well as the production of the inflammatory cytokines. The results showed that LPS treatment induced higher levels of MPO and ROS in cells, whereas Tanshinone IIA coincubation resulted in decreased MPO and ROS levels (Figures 6(a) and 6(b)). In addition, Tanshinone IIA had a remarkable inhibition of cytokines in these cells (Figure 6(c)). Collectively, these data indicated that Tanshinone IIA could inhibit migration and activation of neutrophils in vitro, which were consistent with the results of the in vivo experiments.

## 4. Discussion

The objective of this study was to determine whether Tanshinone IIA could reduce disease in a murine model of colitis through acting specifically on neutrophils and thus could have potential as a therapy in clinical practice. IBD arises from a complex interplay of genetic, microbial, and environmental factors and comprises two major forms: ulcerative colitis (UC) and Crohn's disease (CD) [1, 2, 20]. In the light of UC, it is mostly characterized by recurrent intestinal inflammation, often associated with abdominal pain, diarrhea, blood in the stool, and weight loss [2, 21]. It is recognized that failed inflammation resolution is involved in the pathogenesis of UC. The relevance of dysregulated neutrophil removal from tissues and failed inflammation resolution has been validated [11, 18, 22]. Mechanistically, neutrophils are the first recruited effectors of the acute inflammatory response which defend human hosts from invading microorganisms or tissue injury [7]. However, excessive or persistent accumulation of neutrophils could bring about undesirable outcomes. Recent studies suggest that the robust and continuous influx of neutrophils to the inflamed tissues can cause intestinal mucosal damage by releasing various inflammatory mediators such as ROS and proinflammatory cytokines, and intestinal mucosal damage further stimulates the infiltration of neutrophils, thus forming a vicious circle [21, 23]. Therefore, the powerful effector functions of neutrophils should be tightly controlled to avoid overwhelming or persistent inflammation. However, conventional approaches to neutrophil-targeted anti-inflammatory therapy have their drawbacks. For instance, antibodies or chemokine antagonists that are designed to block neutrophil migration from the vasculature into the tissues or to completely clear neutrophils may impair host defense against infection and lead to the unexpectedly detrimental outcomes [7]. Therefore, it is highly desirable to develop the approaches that are capable of inhibiting overactivation of neutrophils while leaving their essential host-protective functions intact.

Recently, Robertson et al. showed that Tanshinone IIA, a compound derived from a Chinese medicinal herb, potently accelerated inflammation resolution by inducing neutrophil apoptosis and promoting neutrophil reverse migration [12]. This interesting finding may uncover a novel strategy in treating inflammation. Indeed, Tanshinone IIA has been shown to be effective in a number of inflammatory disease models, including LPS-induced lung injury in mice. Tanshinone IIA is reported to have various biological activities, including antioxidative, antifibrotic, antimutagenic, and anticarcinogenic properties [12, 14, 24]. The effects of Tanshinone IIA in inflammatory disease models are suggested to be dependent on the inhibition of the transcription factors such as nuclear factor-*κ*B (NF-*κ*B), activating protein 1 (AP-1), and signal transducer and activator of transcription 1 (STAT1) [12, 13] but have not been attributed to the actions on a single cell type. The pharmacological effects of Tanshinone IIA that have just been identified on neutrophils may play a distinctive role in its protective effect against inflammatory related diseases.

DSS is a heparin-like polysaccharide that has been widely used to induce both acute and chronic colitis in animals [15, 25]. This model exhibits several characteristics resembling human UC, including weight loss, severe diarrhea, rectal bleeding, ulceration and loss of epithelium, and leukocyte infiltration predominantly in the distal colon. In the present study, we adopted the DSS-induced colitis model to assess the protective effects of Tanshinone IIA in mice. Our data showed that the recruitment and activation of neutrophils were prominent in colon mucosa of DSS-treated mice and were associated with the upregulation of ROS and high levels of inflammatory cytokines in the tissues, which were most likely to be involved in disease initiation and progression. Administration of Tanshinone IIA significantly ameliorated the clinical hallmarks of colitis in mice. Furthermore, our data indicated that there was a drastic reduction of neutrophil infiltration and activation in colonic tissues of DSS plus Tanshinone IIA-treated mice, accompanied with decreased ROS and inflammatory cytokine levels, which might account for the efficacy of Tanshinone IIA against intestinal inflammation.

To further consolidate the effect of Tanshinone IIA in modulating neutrophils to block inflammation cascades, we tested the functions of Tanshinone IIA in inhibiting CXCR1-induced neutrophil migration in vitro. Likewise, we examined the effects of Tanshinone IIA in inhibiting LPS-stimulated neutrophil activation, as assessed by the decreased production of MPO, ROS, and inflammatory cytokines. All results from in vitro assays were consistent with in vivo experiments.

Taken together, the data presented here supports that Tanshinone IIA can be an attractive alternative for treatment of colitis by modulation of neutrophils. Novel insights for the anti-inflammatory effects of Tanshinone IIA suggest that it may be applied in the prevention and treatment of various neutrophilic inflammatory diseases.
